# BMP2K dysregulation promotes abnormal megakaryopoiesis in acute megakaryoblastic leukemia

**DOI:** 10.1186/s13578-020-00418-y

**Published:** 2020-04-15

**Authors:** Manman Wang, Tan Zhang, Xuechun Zhang, Zhou Jiang, Min Peng, Zan Huang

**Affiliations:** 1 College of Life Sciences, Wuhan University, No. 299 Bayi Road, Wuhan, Hubei 430072 People’s Republic of China; 2 Department of Oncology, Renmin Hospital of Wuhan University, No. 238 Jiefang Road, Wuhan, Hubei 430060 People’s Republic of China

**Keywords:** BMP2K, AMKL, Megakaryocytes, Polyploidization, Differentiation, CDK2

## Abstract

**Background:**

Forced polyploidization is an effective strategy for acute megakaryoblastic leukemia (AMKL) therapy and factors controlling polyploidization are potential targets for drug development. Although bone morphology protein 2-inducible kinase (BMP2K) has been implied to be a potential target for fasudil, a potent polyploidy-inducing compound, the function of BMP2K in megakaryopoiesis and AMKL remains unknown. This study aimed to investigate the role of BMP2K as a novel regulator in megakaryocyte polyploidization and differentiation and its implication in AMKL therapy.

**Results:**

BMP2K upregulation was observed in human megakaryopoiesis and leukemia cells whereas BMP2K was downregulated in AMKL cells forced to undergo terminal differentiation. Functionally, BMP2K suppressed MLN8237-induced megakaryocytic differentiation in AMKL cells and dampened megakaryocyte differentiation in primary mouse fetal liver cells. Furthermore, BMP2K overexpression conferred resistance to multiple chemotherapy compounds in AMKL cells. Mechanistically, cyclin-dependent kinase 2 (CDK2) interacted with BMP2K and partially mediated its function. In transient MLN8237 and nocodazole challenge cell model, BMP2K reduced cell percentage of G2/M phase but increased G1 phase, suggesting a role of BMP2K antagonizing polyploidization and promoting mitosis by regulating cell cycle in megakaryopoiesis.

**Conclusions:**

BMP2K negatively regulates polyploidization and megakaryocyte differentiation by interacting CDK2 and promoting mitosis in megakaryopoiesis. BMP2K may serve as a potential target for improvement of AMKL therapy.

## Introduction

Polyploidization and functional maturation are two accompanying and distinct processes of megakaryopoiesis [1]. Megakaryocyte polyploidization increases DNA content and cytoplasmic mass and promotes organelle development such as marcation membrane system that promotes the efficiency of platelet production. Defects in polyploidization are associated with many human pathologies. For instance, increased hypoploid megakaryocytes are present in myelodysplastic syndromes (MDS), which is one of important diagnostic criteria for myeloproliferative neoplasms (MPNs) [2]. Particularly, acute megakaryoblastic leukemia (AMKL) cells display diploidy due to differentiation blockage at promegakaryoblast stage and lose the ability to undergo polyploidization [1]. Although multiple factors including transcription factors, signal transduction pathways, epigenetic modifiers, cell cycle regulators affect polyploidization, the underlying mechanisms remain to be addressed.

Endomitosis is the cause of polyploidization in megakaryocytes, which involves several successive rounds of DNA replication and is composed of repeated G1, S, G2, M phase. However, the endomitotic megakaryocytes skip cytokinesis due to regression of cleavage furrow resulting in cells that contain multiple copies of a normal diploid chromosome content [3, 4]. Cell cycle regulation is believed to be the key to induce polyploidization and differentiation in megakaryocytes and AMKL cells. RhoA pathway is a critical regulator of cleavage furrow formation and ingression in mitosis. Deficiency in RhoA pathway activation that results in failure of cleavage furrow formation is essential for endomitosis [4]. Therefore, targeting RhoA pathway to induce polyploidization has been proposed to be an interesting therapeutic approach for AMKL [5]. MLN8237 (Alisertib), a selective inhibitor of aurora kinase A (AURKA), has also been reported to induce polyploidization and the expression of mature megakaryocyte markers in AMKL and PMF blasts by promoting the transition from the proliferative cell cycle to an endomitosis [5, 6]. Although the exact mechanism determining whether megakaryocytes undergo mitosis or endomitosis remains unresolved, identification of pivotal polyploidy-inducing regulators may help address this issue and provide potential targets for AMKL therapy.

Bone morphology protein 2-inducible kinase (BMP2K) has been implied as a potential regulator in megakaryocyte polyploidization [5]. BMP2K was originally identified as a BMP2-induced serine/threonine protein kinase during osteoblast differentiation [7, 8]. BMP2K was also correlated to the developmental dysplasia of the hip [9] and susceptibility of high myopia [10]. BMP2K may confer resistance to fludarabine in Chronic Lymphocytic Leukemia (CLL) [11]. Other reports suggest that BMP2K is a potential regulator of blood stem/progenitor cells [12]. Although BMP2K has been implied to be a potential target for fasudil, a potent polyploidy-inducing compound, the function of BMP2K in megakaryopoiesis remains unknown.

In this study, we showed that BMP2K was associated with normal and malignant megakaryopoiesis. BMP2K functioned as a negative regulator of megakaryocyte differentiation in AMKL cells and primary mouse fetal liver cells and BMP2K upregulation contributed to resistance to multiple compounds in AMKL cells. Mechanism study showed that BMP2K suppressed megakaryocyte differentiation by interacting CDK2 and antagonizing polyploidy-inducing conditions and maintaining AMKL cells in mitosis. Thus, our work offers new insights into the role of BMP2K in polyploidization and differentiation of megakaryocytes and AMKL cells.

## Results

### BMP2K is involved with normal and malignant megakaryopoiesis

**Fig. 1:**
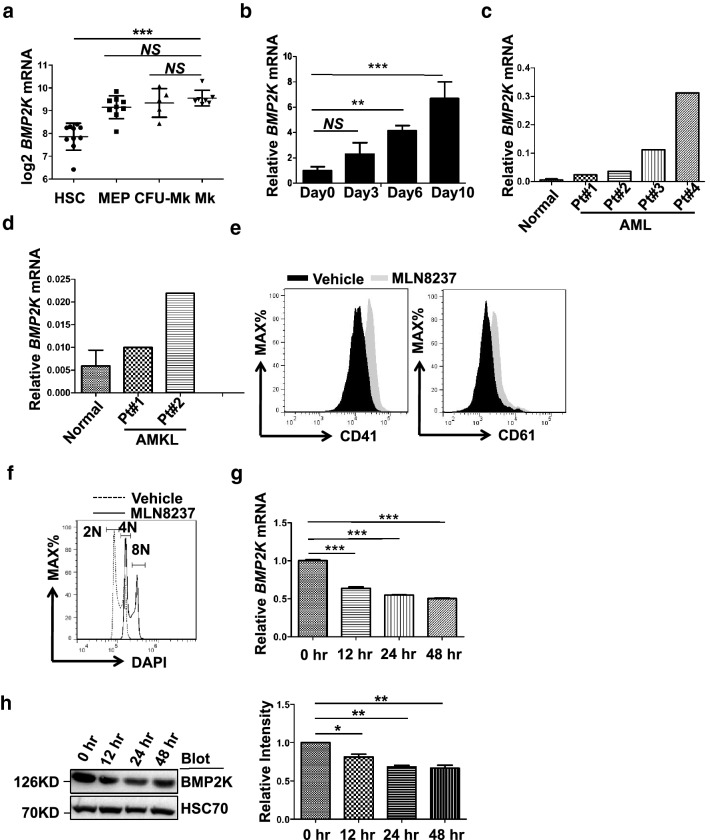
BMP2K is involved with normal and malignant megakaryopoiesis. **a** Public database (BloodSpot) shows upregulation of BMP2K in megakaryocytes. **b** Human CD34^+^ HSC were cultured under megakaryocyte differentiation medium for different days as indicated. The upregulation of *BMP2K* mRNA was confirmed by quantitative RT-PCR. **c**–**d** Upregulation of *BMP2K* mRNA in mononuclear cells isolated from AML (**c**) and AMKL (**d**) patients was confirmed by quantitative RT-PCR compared with that from peripheral blood of heathy (Normal, n = 7) donors. **e**–**f** CMK cells were induced to undergo megakaryocytic differentiation by MLN8237 (1 μM) treatment for 48 h, which was measured by staining the surface markers CD41 and CD61 (**e**) or staining the DNA with DAPI (**f**). Gates represent cells with diploidy (2 N), tetraploidy (4 N) and octaploidy (8 N). **g** Downregulation of *BMP2K* mRNA in CMK cells undergoing MLN8237-induced megakaryocytic differentiation for different hours was confirmed by quantitative RT-PCR. **h** The BMP2K protein in MLN8237-treated cells was also measured by immunoblotting. The right panel is the statistics of the densitometric analysis of the immunoblotting. * *p* < 0.05, ** *p* < 0.01; *** *p* < 0.001; *NS*, non-significance for the comparison as indicated

Previous studies suggest that BMP2K may be a potential regulator of blood stem/progenitor cells [12] as well as AMKL [5]. Indeed, the public database (Bloodspot, https://servers.binf.ku.dk/bloodspot/) showed upregulation of BMP2K in megakaryocyte lineage compared to hematopoietic stem cell (HSC) (Fig. 1a). Consistently, BMP2K expression increased over time when human CD34^+^ cells were cultured under megakaryocyte differentiation conditions (Fig. 1b). Interestingly, we also observed upregulation of BMP2K in primary human peripheral mononuclear cells isolated from AML and AMKL patient samples compared to that in healthy donor samples (Fig. 1c, d). Notably, MLN8237 as a potent inhibitor of Aurora A kinase that promoted the expression of markers of megakaryocytic differentiation (CD41 and CD61) and polyploidization in CMK cells (Fig. 1e, f) also caused significant BMP2K downregulation at both mRNA and protein levels (Fig. 1g, h). These observations demonstrate that BMP2K is involved with normal and malignant megakaryopoiesis.

### BMP2K antagonizes MLN8237-induced megakaryocytic differentiation in CMK cells

**Fig. 2:**
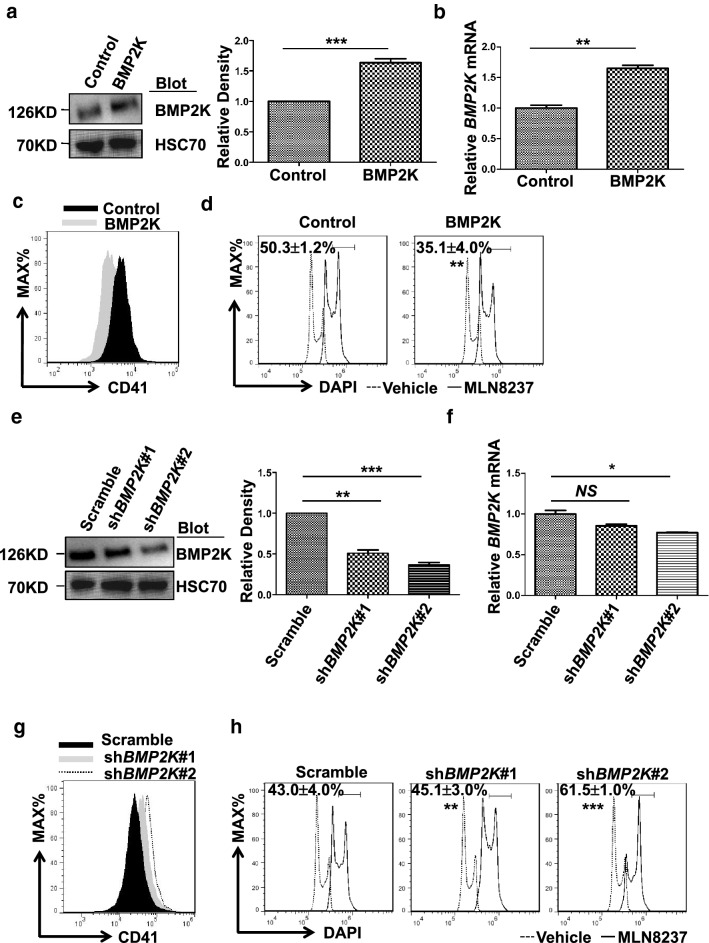
BMP2K inhibits MLN8237-induced megakaryocytic differentiation. a BMP2K protein level in Control and BMP2K overexpression cells was confirmed by immunoblotting (left panel). The right panel is the statistics of the densitometric analysis of the immunoblotting. **b***BMP2K* mRNA level in these cells was measured by quantitative RT-PCR. **c**–**d** Control and BMP2K overexpression cells were treated with Vehicle (dot line) or MLN8237 (solid line) for 48 h. The megakaryocytic differentiation was measured by CD41 staining (**c**) or by DAPI staining for DNA content (**d**). **e**–**h** BMP2K downregulation by shRNAs (sh*BMP2K*#1 and sh*BMP2K*#2) in CMK cells and its positive effect on MLN8237-induced megakaryocytic differentiation was measured as described in **a**–**d**. Gates in D and H represent cells with ploidy ≥ 8 N. * *p* < 0.05, ** *p* < 0.01; *** *p* < 0.001; *NS*, non-significance compared with Control or Scramble cells

To determine the function of BMP2K in AMKL, we took advantage of the CMK cell model. We overexpressed BMP2K in CMK cells through lentiviral transduction (Fig. 1a, b) and induced megakaryocytic differentiation by using MLN8237. BMP2K overexpression impaired MLN8237-induced megakaryocytic differentiation evidenced by the reduced expression level of CD41 and the decreased the percentage of polyploidy cells (≥ 8N) compared to that in control cells (Fig. 2c, d). Furthermore, we designed two individual shRNAs (sh*BMP2K*#1 and#2) specifically targeting human *BMP2K* gene. Both shRNAs significantly decreased BMP2K protein level at least by half despite that *BMP2K* knockdown at mRNA level was not as potent as protein level (Fig. 2e, f). Notably, both shRNAs enhanced MLN8237-induced megakaryocytic differentiation (Fig. 2g, h), which demonstrated the specificity of these two shRNAs. Particularly, sh*BMP2K*#2 appeared to be more efficient than sh*BMP2K*#1 in downregulating BMP2K, which was consistent to more potent effect of sh*BMP2K*#2 than that of sh*BMP2K*#1 on promoting polyploidization and CD41 expression. Therefore, we used sh*BMP2K*#2 for further experiments. These results imply that BMP2K is a negative regulator of megakaryocyte differentiation.

### BMP2K alters cell cycle in megakaryocytes in response to MLN8237 challenge

**Fig. 3:**
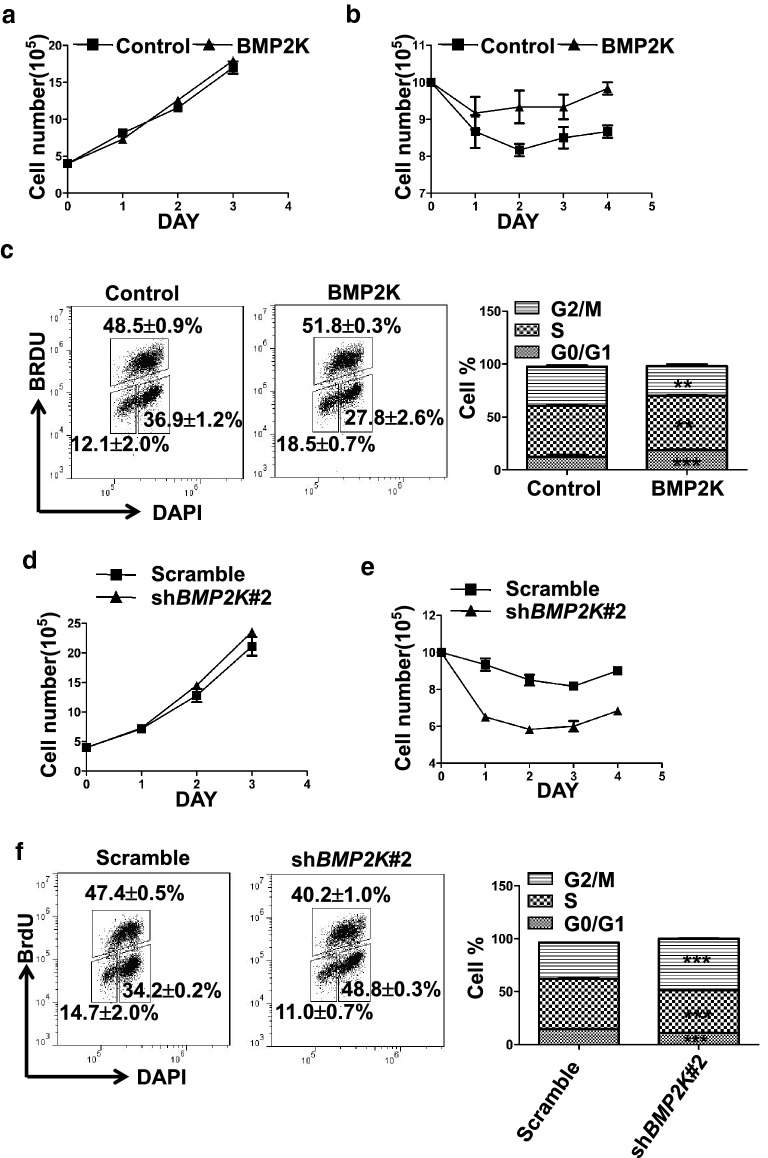
BMP2K promotes proliferation in response to MLN8237 challenge. Control or BMP2K overexpression cells were cultured in basal conditions (**a**) or in the presence of MLN8237 (0.5 μM) for 6 h followed by wash and reseeding in fresh medium (**b**) for a consecutive 3 days. The cell numbers were counted on each day for cell proliferation assay. **c** The MLN8237-treated cells were further labeled with BrdU and harvested for cell cycle profile analysis by staining BrdU with a fluorescence-conjugated BrdU antibody and staining DNA with DAPI. The right panel is the statistics of the cell cycle profile. **d**–**f** The positive effect of BMP2K knockdown (sh*BMP2K*#2) on cell proliferation were detected and cell cycle profile was analyzed as described in A-C. * *p* < 0.05, ** *p* < 0.01; *** *p* < 0.001; *NS*, non-significance compared with Control or Scramble cells

Cell cycle regulation is key to polyploidization. Therefore, we investigated whether BMP2K affected cell cycle in megakaryocyte differentiation. Apparently, BMP2K overexpression did not affect CMK cell proliferation under regular culture conditions (Fig. 3a). Alternatively, we challenged CMK cells with MLN8237 for 6 h and re-seeded these cells in fresh medium without MLN8237. Transient MLN8237 treatment may be sufficient to push cell progress to G2/M phase but not enough to achieve polyploidy in CMK cells. Upon MLN8237 withdrawal, these cells may return to mitosis. We found that more BMP2K overexpression cells recovered from a transient MLN8237 challenge than control cells over 4 days culture (Fig. 3b). Indeed, PI staining analysis showed that the BMP2K overexpression cells displayed less cells at G2/M phase but more cells at G1/G0 and S phase than control cells (Fig. 3c). We further examined the effect of BMP2K downregulation. Again, BMP2K knockdown did not alter CMK cell proliferation at regular culture conditions (Fig. 3d). However, BMP2K knockdown significantly reduced cells that recovered from MLN8237 challenge, increased cells at G2/M phase but decreased cells at G1/G0 and S phase (Fig. 3e, f). These observations demonstrate that BMP2K alters cell cycle in megakaryocytes under polyploidy-inducing conditions.

### CDK2 mediates BMP2K function in MLN8237-induced megakaryocytic differentiation

**Fig. 4:**
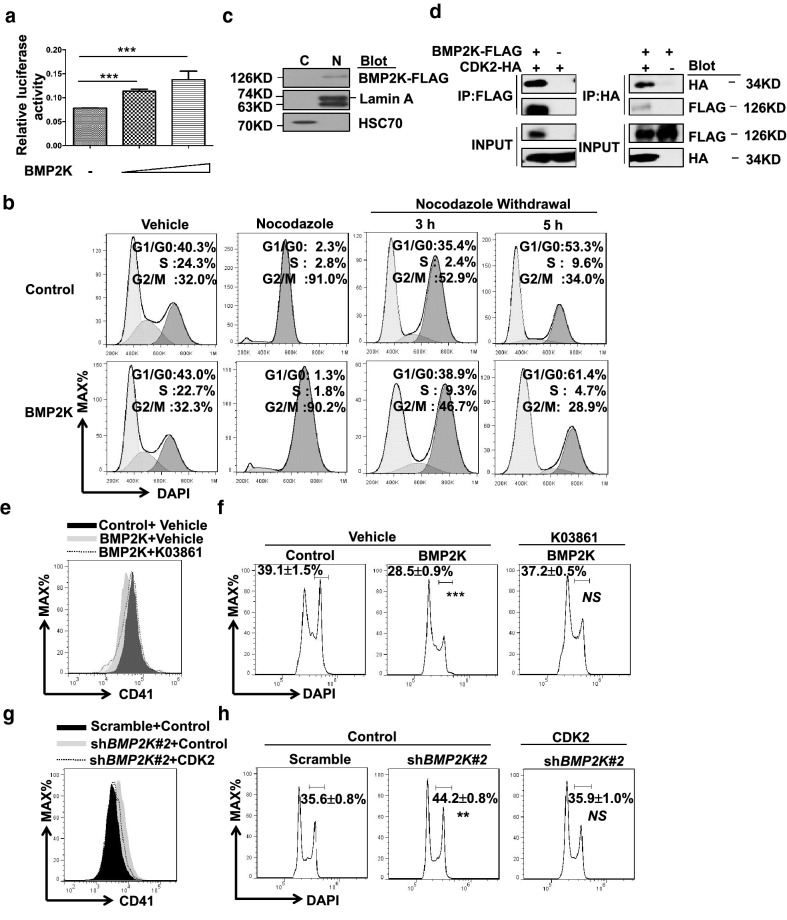
CDK2 mediates BMP2K function in MLN8237-induced megakaryocytic differentiation. **a** The luciferase activity of an E2F-responsive element in 293T cells co-transfected without (-) or with an increased amount (triangle) of BMP2K overexpression vector. ***, *p* < 0.001 for comparison as indicated. **b** Control and BMP2K overexpression cells were treated with Nocodazole (10 ng/ml) for 6 h followed by wash and culturing in fresh medium for 3 or 5 h as indicated (Nocodazole Withdrawal). The cell cycle profile was measured by DAPI staining followed by flow cytometry analysis. **c** The presence of BMP2K in the nuclear extract (N) from BMP2K overexpression cells was detected by immunoblotting. Lamin A and HSC70 serve as positive proteins for nuclear extract and cytosolic extract (**c**), respectively. **d** The absence or presence of BMP2K-FLAG and CDK2-HA expression plasmids were indicated as – or +, respectively. Plasmids with different combinations were transfected into 293T cells for co-immunoprecipitation with FLAG antibody (IP: FLAG, left panel) or with HA antibody (IP: HA, right panel). The BMP2K-FLAG and CDK2-HA in IP and lysates (INPUT) was detected by immunoblotting. **e**–**f** MLN8237 together with Vehicle or CDK2 inhibitor K03861 (10 nM) as indicated induced Control and BMP2K overexpression cells undergoing megakaryocytic differentiation, which was evaluated by CD41 staining (**e**) and DAPI staining for DNA content (**f**). **g**–**h** CDK2 overexpression in sh*BMP2K#2* cells (sh*BMP2K#2 *+ CDK2) as indicated offset the effect of BMP2K deficiency on MLN8237-induced megakaryocytic differentiation, which was measured by CD41 straining **g** and DAPI staining for DNA content **h** Gates in F and H represent cells with ploidy ≥ 8 N. * *p* < 0.05, ** *p* < 0.01; *** *p* < 0.001; *NS*, non-significance compared with Control or Scramble cells

Cell cycle alteration in BMP2K overexpression cells under MLN8237 challenge conditions suggests that BMP2K may play a role in G1-S progress or G2/M exit. Indeed, BMP2K overexpression increased the luciferase activity of a promoter driven by an E2F responsive element (Fig. 4a). E2F is known to promote G1-S transition and progress and our findings suggest a potential role of BMP2K in G1-S progress. We also used nocodazole to arrest cell at G2/M phase and examined whether BMP2K promoted the exit of these cells from G2/M and return to G1 phase upon Nocodazole withdrawal. Nocodazole treatment caused cell cycle arrested at G2/M phase in both control and BMP2K overexpression cells (Fig. 4b). Upon nocodazole withdrawal, BMP2K overexpression cells showed modest but consistent increase of cells in G1/G0 phase and decrease of G2/M phase over time (Fig. 4b). These observations suggest that BMP2K may regulate cell cycle by promoting megakaryocytes to exit G2/M phase and return to G1 phase.

To explore the underlying mechanism that BMP2K regulates cell cycle in megakaryocyte differentiation, we analyzed the subcellular localization of BMP2K and showed that BMP2K was present in nuclear fraction (N) but not cytosolic fraction (C) (Fig. 4c). Public database (Biogrid, https://thebiogrid.org/) search suggests that cyclin-dependent kinase 2 (CDK2) is a potential interacting partner of BMP2K. CDK2 drives both G1-S-phase transit and S-phase progression, which is consistent to our findings showing the effect of BMP2K on enhancing luciferase activity of an E2F-reponsive element (Fig. 4a). Indeed, we confirmed BMP2K interaction with CDK2 by co-immunoprecipitation followed by immunoblotting (Fig. 4d). Furthermore, we tested whether CDK2 was required for BMP2K function in megakaryocyte differentiation. We used CDK2 inhibitor K03861 [13] in BMP2K overexpression cells (BMP2K + K03861) to abrogate the CDK2 activity. Upon MLN8237 treatment, BMP2K overexpression cells (BMP2K + Vehicle) showed impaired megakaryocytic differentiation compared to control cells (Control + Vehicle). Notably, K03861 restored CD41 expression and rescued the impaired polyploidization in BMP2K overexpression cells (BMP2K + K03861) compared to BMP2K cells without K03861 (BMP2K + Vehicle) (Fig. 4e, f). In contrast, BMP2K knockdown (sh*BMP2K*#2 + Control) promoted MLN8237-induced megakaryocytic differentiation whereas CDK2 overexpression in BMP2K knockdown cells (sh*BMP2K*#2 + CDK2) potently neutralized CD41 expression and offset the enhanced polyploidization comparable to scramble cells (Scramble + Control) (Fig. 4g, h). These findings suggest that CDK2 may be required for and sufficient to mediate BMP2K function in MLN8237-induced megakaryocytic differentiation.

### BMP2K inhibits normal megakaryopoiesis in primary mouse fetal liver cells

**Fig. 5:**
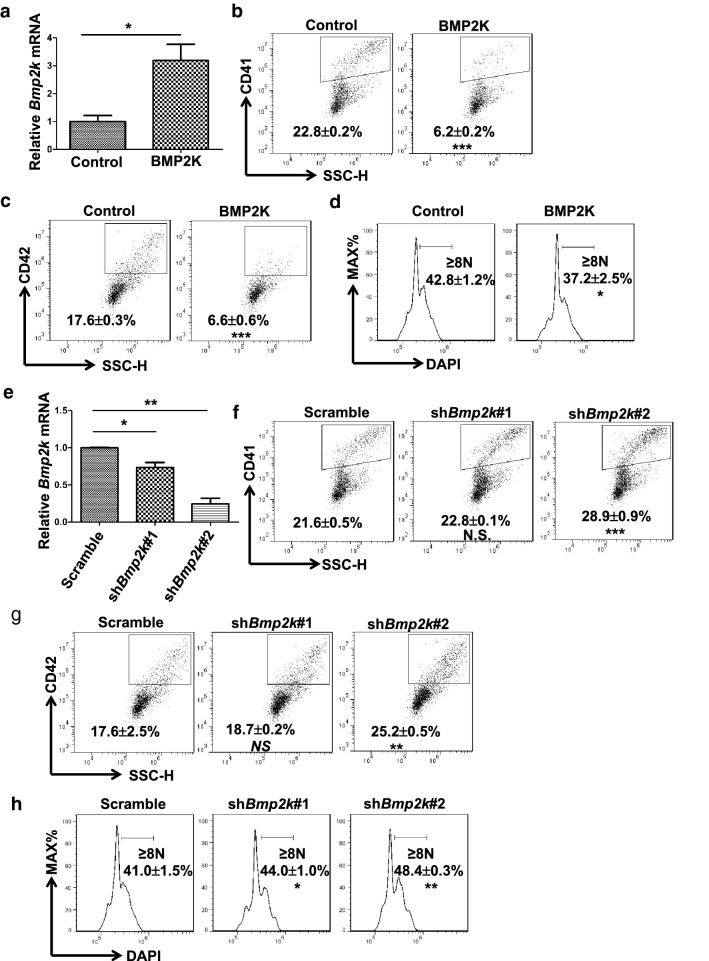
BMP2K suppresses megakaryopoiesis in primary mouse fetal liver cells. **a** Mouse fetal liver cells were transduced with Control or BMP2K overexpression retrovirus. The expression level of BMP2K mRNA was measured by quantitative RT-PCR. **b**–**d** The transduced fetal liver cells were induced to undergo megakaryocyte differentiation for 3 days. The megakaryocyte differentiation was measured by staining for CD41 (**b**), CD42 (**c)** and DNA content (**d**). **e**–**h** The downregulation of BMP2K and its effect on megakaryopoiesis was performed as described in (**a**–**d**). Gates in B, C, F, G represent CD41 or CD42 positive cells as indicated and gates in D and H represent cells with ploidy ≥ 8 N. * *p* < 0.05, ** *p* < 0.01; *** *p* < 0.001; *NS*, non-significance compared with Control cells or Scramble cells

We further tested BMP2K function on normal megakaryopoiesis in primary mouse fetal liver cells. Wildtype E12.5 fetal liver cells were harvested and transduced with retrovirus expressing BMP2K or shRNAs specific for mouse BMP2K (sh*Bmp2k*#1, sh*Bmp2k*#2). Three days after selection and differentiation in the presence of TPO, the megakaryocyte differentiation was evaluated by measuring CD41 and CD42 expression as well as DNA content. Consistently, BMP2K overexpression (Fig. 5a) suppressed CD41 and CD42 expression and reduced polyploidization in CD41 positive cells (Fig. 5b–d). In contrast, BMP2K knockdown (Fig. 5e) promoted CD41 and CD42 expression as well as polyploidization (Fig. 5f–h). These observations suggest that BMP2K may be a negative regulator in normal megakaryopoiesis.

### BMP2K overexpression confers resistance to multiple chemotherapy compounds in AMKL cells

**Fig. 6:**
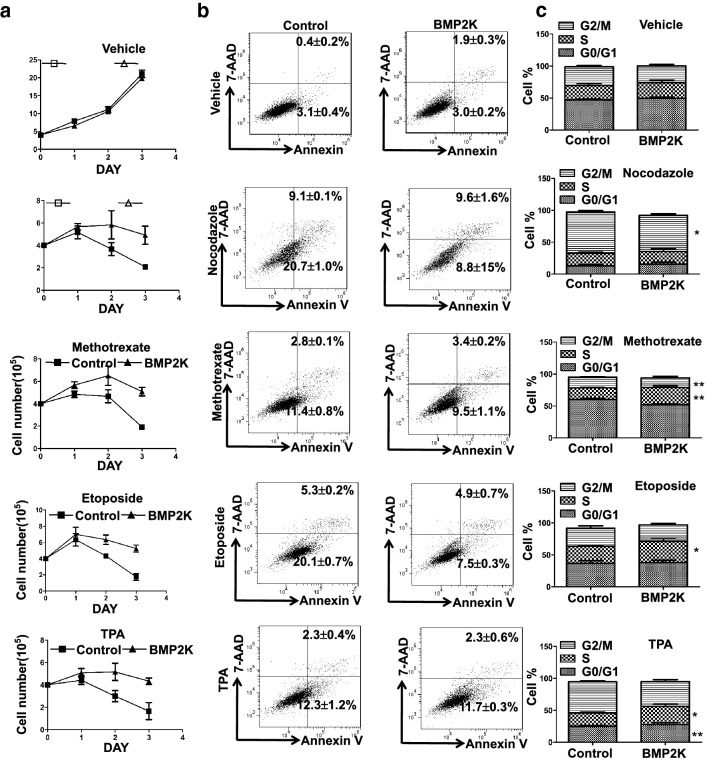
BMP2K overexpression confers AMKL cells of resistance to multiple chemotherapy compounds. Control and BMP2K overexpression CMK cells were cultured with Vehicle or chemotherapy compounds (10 ng/ml Nocodazole, 2.5 nM Methotrexate, 50 nM TPA and 10 μM Etoposide) for 3 days. The cell numbers were counted on each day for cell proliferation assay **a** Apoptosis was measured on day 1 by Annexin V and 7-AAD staining and analyzed by flow cytometry **b** The cell cycle profile was also measured by PI staining and analyzed by flow cytometry on day 1 **c** * *p* < 0.05, ** *p* < 0.01; *** *p* < 0.001; *NS*, non-significance compared with Control cells

AMKL resistance to multiple chemotherapy is one important factor leading to dismal prognosis. The effect of BMP2K on antagonizing MLN8237 suggest that BMP2K overexpression may contribute to the chemotherapy resistance in AMKL. BMP2K was previously suggested to confer resistance to fludarabine in Chronic Lymphocytic Leukemia. Therefore, we examined whether BMP2K overexpression contribute to the resistance to multiple chemotherapy compounds including Nocodazole, Methotrexate, Etoposide, and TPA in AMKL. Nocodazole can induce polyploidization of AMKL cell line more dramatic than TPA and effectively block megakaryocytes cultured from Human CD34^+^ into typical pseudo-metaphase state [14, 15]. Methotrexate is used for therapy of ALL and AMKL [16, 17]. Etoposide triggers mitochondrial damage, caspase activation and cell death in megakaryocytes [18] and induce genotoxic stress by activating the Hippo-p53 axis in megakaryocytes [19]. All these compounds efficiently suppressed proliferation of control cells whereas BMP2K overexpression antagonized the effect of all these compounds and improved cell proliferation (Fig. 6a). BMP2K reduced apoptosis in Nocodazole- and Etoposide-treated cells whereas it did not alter apoptosis in Methotrexate- and TPA-treated cells (Fig. 6b). BMP2K also increased cells in S phase in Methotrexate-, Etoposide-, and TPA-treated cells (Fig. 6c). These observations suggest that BMP2K upregulation may be an important mechanism causing resistance to chemotherapy in AMKL. BMP2K may antagonize the effect of multiple chemotherapy compounds by improving apoptosis and/or cell cycle arrest.

## Discussion

Inducing megakaryocyte polyploidization is a novel strategy for therapy of AMKL and MPNs. Study on the factors contributing to polyploidization may provide potential targets for this purpose. BMP2K has been implied as a potential target of polyploidy-inducing compound fasudil in previous study [5]. However, the function of BMP2K is unknown and its role in megakaryopoiesis has not been studied. In this study, we showed BMP2K as a negative regulator of megakaryopoiesis and played important functions in AMKL.

We discovered three important phenomena of BMP2K in regulating polyploidization of CMK cells and primary mouse fetal liver cells: First, BMP2K may block the transfer from proliferation (or mitosis) to differentiation (or polyploidization) since downregulation of BMP2K resulted in an increased number of polyploid cells (Fig. 2f). Second, BMP2K affects cell cycle in AMKL cells undergoing megakaryocytic differentiation. G1/G0 and S phase cells are increased in BMP2K overexpression CMK cells under MLN8237 challenge and BMP2K deficiency displayed opposite phenotype (Fig. 3c, d). Third, BMP2K interacts and co-localizes with CDK2 and may functionally rely on CDK2 (Fig. 4).

Endomitosis is the mechanism of megakaryocyte polyploidization and cell cycle regulation is the key to induced endomitosis. Multiple cyclins, cyclin-dependent kinases, cell cycle regulators, chromosome passenger proteins are proposed to play critical roles [1, 5, 15, 20]. For instance, high expression level of cyclin D 1 and D3 and deficiency of CDK inhibitor p19^INK4D^ increase polyploidization [21–24]. On the other hand, some key kinases in cell cycle regulation play important role in mitosis, but are dispensable for megakaryocytes maturation, such as AURKA, CDK1, CDK2, cell division cycle 20 (CDC20). However, inhibitors of these kinases are potent inducers of polyploidization. AURKA is an essential negative regulator of polyploidization in AMKL blasts [5, 25] and has been proposed as a target for AMKL therapy. The selective inhibition of AURKA activity by AURKA inhibitor (MLN2837) significantly induced polyploidization of AMKL cell [5]. CDK2 also plays an important role in regulating both G1-S-phase transit and S-phase progression. A number of regulatory cyclins are complexed with CDK2 in these processes, like cyclin E and cyclin A, which are required for CDKs kinase activity [26–30]. In our study, we found that BMP2K interacted with CDK2 and activated an E2F-responsive element. Moreover, CDK2 is required for BMP2K function in MLN8237-induced megakaryocytic differentiation (Fig. 4). These results emphasize that there may exist an AURKA-BMP2K-CDK2 axis promoting megakaryocytes reentry into mitosis. However, the effects of loss of AURKA or BMP2K on megakaryocytes may not simply stem from inhibition of the proliferative cell cycle, as other cell cycle inhibitors known to induce polyploidy failed to induce differentiation of the megakaryocyte lineage by itself, including AURKB, ROCK1, CDK1, and PLK1 [25]. These observations imply that except for cell cycle regulation, BMP2K might function through other regulatory mechanisms to involve in megakaryocyte differentiation.

Our study also highlights a potential role of BMP2K in AMKL therapy. Resistance to multiple chemotherapy is one important factor resulting in dismal prognosis of AMKL. Although MLN8237 is under clinical investigation for AML, the patients lacking the AURKA subtype limit the application of MLN8237 in target therapy of AMKL [5, 31]. In this study, the effect of BMP2K on antagonizing MLN8237 suggest that BMP2K overexpression may contribute to the chemotherapy resistance in AMKL. In fact, we showed that BMP2K overexpression antagonized multiple chemotherapy compounds that were known to cause megakaryocyte differentiation (nocodazole and TPA) or apoptosis (etoposide) [14, 15, 18, 32] or use for therapy of ALL and AMKL (methotrexate) [16, 17]. Our findings suggest that BMP2K upregulation may be an important mechanism causing drug resistance in AMK. These results also further rationalize the potential application of BMP2K inhibitor to improve chemotherapy. CDK2 overexpression is known to cause drug resistance in AMKL. Whether BMP2K overexpression depends on CDK2 to cause drug resistance remains to be explored. Nevertheless, inhibition of BMP2K may be an auxiliary way to enhance the efficacy of chemotherapy. BMP2K inhibitor combination with a variety of other chemotherapy reagents may be an interesting therapeutic strategy for AMKL.

## Conclusion

Our observations emphasize an important role of BMP2K that may promote megakaryocytes reentry into mitosis and functioned as a negative regulator of megakaryocyte polyploidization and differentiation. Subsequently, it will be interesting to investigate on how BMP2K be involved in the kinase network controlling the transfer from a proliferative cell cycle to a polyploid, which provides scientific basis for targeted therapy of AMKL.

## Materials and methods

### Cell culture, animals and blood samples

The human leukemia cell lines CMK were cultured in a complete 1640 RPMI medium and HEK293T cells were maintained in a complete Dulbecco’s modified Eagle medium (Gibco BRL, Grand Island, NY, USA), both of which were supplemented with 10% fetal bovine serum, 1% streptomycin and penicillin. CMK cells treatment with Nocodazole (10 ng/ml), Methotrexate (2.5 nM), Etoposide (10 μM), and TPA (50 nM) was carried out as previously described [14, 18, 33–35]. Human megakaryocyte culture experiments were performed by culturing CD34^+^ cells (purchased from Fred Hutchinson Cancer Research Center, USA) in StemSpam SFEM media (Stemcell Technologies, Vancouver, BC, Canada) supplemented with penicillin/streptomycin and lipids (40 mg/ml) as well as megakaryocyte differentiation cytokine cocktail containing stem cell factor (100 ng/ml) and TPO (10 ng/ml) for 10 days [35, 36]. Culturing mouse megakaryocytes from fetal liver cells was carried out as previously described [37].

All animal studies were approved by the Animal Care and Use Committees of College of Life Sciences of Wuhan University. Mononuclear cells (MNCs) were isolated from peripheral blood samples on Ficoll-Hypaque density gradients. All experiments involving human blood samples were approved by the Medical Ethics Committees of Renmin Hospital of Wuhan University. Consent form was obtained from each patient and healthy donor.

### Quantitative RT-PCR

Quantitative RT-PCR (qRT-PCR) analysis was performed according to our previous report [37]. TRIzol reagent (Invitrogen, Grand Island, NY, USA) was used to extract total RNA. MonScript™ RTIII Super Mix with dsDNase Kit (Monad, Wuhan, China) was used to reverse-transcribe RNA into complementary DNA. Quantitative RT-PCR was performed in the following conditions: hot-start at 95 °C for 60 s followed by 95 °C for 30 s, 60 °C for 30 s for 40 cycles. The reactions were run on Fast Real-Time PCR System (Monad, Suzhou, China). The relative quantitation of real-time PCR was determined using the comparative ∆Ct method and presented in a bar graph format and GAPDH serves as a control for normalization. The primer sets used for the qRT-PCR analysis are as following: human-*BMP2K* forward: ACCAAAGGCCAACTCTGCTAC, human-*BMP2K* reverse: GACCCAATAAAATTTCAGGGCCA; human-*GAPDH* forward: CATCACCATCTTCCAGGAGCGAGA, human-*GAPDH* reverse: TGCAGGAGGCATTGCTGATGATCT; mouse-*Bmp2k* forward: GTCAATAACACACCCGACCTC, mouse-*Bmp2k* reverse: AACCTGCCCAGCTCGACAATA; mouse-*Gapdh* forward: TTTGTCAAGCTCATTTCCTGGTATG, mouse-*Gapdh* reverse: TGGGATAGGGCCTCTCTTGC.

### Immunoprecipitation and Western blot analysis

Immunoprecipitation and Western blotting were performed according to a standard protocol [37]. Antibodies included mouse anti-BMP2K (Santa Cruz Biotechnology, Santa Cruz, CA, USA), mouse anti-HSC70 (Santa Cruz Biotechnology, Santa Cruz, CA, USA), mouse anti-FLAG antibody (Sigma,St Louis, MO, USA), mouse anti-HA (ProteinTech Group, Chicago, IL, USA). Results are representative blot from at least three blots with similar results.

### Flow cytometry analysis of megakaryocyte differentiation

Megakaryocyte differentiation was measured as previously described [35]. CMK cells treated with MLN8237 (1 μM) for 48 h [5] were stained with phycoerythrin-conjugated anti-CD41 or phycoerythrin-conjugated anti-CD61 antibody (BD Biosciences, San Jose, CA, USA) and DAPI. Megakaryocyte differentiation of primary mouse fetal liver cells were stained with phycoerythrin-conjugated anti-mouse CD41, APC-conjugated anti-mouse CD42 antibodies and DAPI. FACS data were analyzed with the FlowJo software (TreeStar, Ashland, OR, USA).

### BrdU staining

BrdU staining was performed as previously described [38]. Briefly, cells were labeled with bromodeoxyuridine (30 μg/ml), fixed (2% paraformaldehyde), permeabilized (400 μl of 150 mM NaCl, 850 μl of 100% ethanol), and fixed (200 μl Hanks balanced salt solution, 250 μl 2% PFA, 50 μl 1%Tween 20) again overnight at 4 °C. Cells were treated with DNase I (50U) in DNase I buffer (40 mM Tris–HCl, 10 mM NaCl, 6 mM MgCl2, 1 mM CaCl_2_, pH7.9) with 50U DNase I per sample in 37 °C for 1 h. After wash, cells were stained with Alexa 647-labeled anti-BrdU antibody (BD Biosciences, San Jose, CA, USA) and DAPI for 1 h at room temperature in dark and analyzed by flow cytometry.

### Lentivirus or retrovirus infection

Gene overexpression or knockdown was achieved through lentiviral or retroviral transduction [38]. Vectors carried puromycin-resistant gene and the transduced cells were selected with puromycin (1 mg/ml) for a week to obtain stable cell lines. Human BMP2K was fused with a Flag tag in the C-terminal. BMP2K shRNA for knocking down human or mouse BMP2K were determined by online shRNA searching tool (https://portals.broadinstitute.o-rg/gpp/public/). The specificity of shRNA was further determined by using NCBI’s BLAST program to minimize the degradation of off-target mRNAs. Only sequences that exhibited at least 3 nucleotide mismatches to all other unrelated genes were selected. We chose and synthesized two top ranking sequences as BMP2K shRNAs and subcloned into a lentiviral expression vector. The sequences of shRNA oligos for human BMP2K are as follows: sh*BMP2K*#1:5′-GACCTTCTAAGATCAAGTAAGCTCGAG-3′; sh*BMP2K*#2:5′-TCTTCTATTCCTTCAGCTCTTCTCGAG-3′. The sequences of shRNA oligos for mouse Bmp2k are as follows: sh*Bmp2k*#1:5′-GGAACATTCTCCAAATCAAACTCGAG-3′; sh*Bmp2k*#2:5′-CCGGTCTCCAACATCAATAATCTCGAG-3.

### Dual luciferase activity assay

Dual luciferase activity assays were performed in 293T cells. Briefly, a firefly luciferase reporter vector driven by a promoter containing an E2F-responsive element was co-transfected with the internal control pRL-TK vector expressing a renilla luciferase in recombination with BMP2K overexpression plasmid or control plasmid. The dual luciferase activity was assayed according to the manufacturer’s instructions (Dual-Luciferase Reporter Assay System, Promega, Madison, WI, USA). The promoter activity was presented as relative luciferase activity by normalizing the firefly luciferase activity to the internal control of renilla luciferase activity.

### Statistical analysis

All statistical analyses were performed using a *Student’s t*-*test* (two-tailed, unpaired) to determine the significance of differences in comparison. P-value of ≤ 0.05 was considered statistically significant. Statistic results of cell proliferation assay, dual luciferase activity assay, qRT-PCR and flow cytometry are derived from the representative experiment with triplicates of at least three independent experiments with similar results. Statistical results of immunoblotting are derived from the densitometric analysis of one representative blot of at least three independent experiments with similar results.

## Data Availability

The data that support the findings of this study are available from the corresponding author upon request.

## Abbreviations

BMP2K: Bone morphology protein 2-inducible kinase
AMKL: Acute megakaryoblastic leukemia
CDK2: Cyclin-dependent kinase 2
HSC: Hematopoietic stem cell
AML: Acute myelocytic leukemia
MKs: Megakaryocyte
qRT-PCR: Quantitative real-time PCR
shRNA: The short hairpin RNA
